# Teaching systems thinking to 4^th^ and 5^th^ graders using Environmental Dashboard display technology

**DOI:** 10.1371/journal.pone.0176322

**Published:** 2017-04-27

**Authors:** Shane Clark, John E. Petersen, Cindy M. Frantz, Deborah Roose, Joel Ginn, Daniel Rosenberg Daneri

**Affiliations:** 1 Environmental Studies Program, Oberlin College, Adam Joseph Lewis Center, Oberlin, Ohio, United States of America; 2 Psychology Department, Oberlin College, Severance Laboratory, Oberlin, Ohio, United States of America; 3 Education, Oberlin College, AJLC Annex, Oberlin, Ohio, United States of America; 4 Department of Natural Resources, Cornell University, Fernow Hall, Ithaca, New York, United States of America; The Hague University of Applied Science, NETHERLANDS

## Abstract

Tackling complex environmental challenges requires the capacity to understand how relationships and interactions between parts result in dynamic behavior of whole systems. There has been convincing research that these “systems thinking” skills can be learned. However, there is little research on methods for teaching these skills to children or assessing their impact. The Environmental Dashboard is a technology that uses “sociotechnical” feedback–information feedback designed to affect thought and behavior. Environmental Dashboard (ED) combines real-time information on community resource use with images and words that reflect pro-environmental actions of community members. Prior research indicates that ED supports the development of systems thinking in adults. To assess its impact on children, the technology was installed in a primary school and children were *passively* exposed to ED displays. This resulted in no measurable impact on systems thinking skills. The next stage of this research examined the impact of *actively* integrating ED into lessons on electricity in 4^th^ and 5^th^ grade. This active integration enhanced both content-related systems thinking skills and content retention.

## Introduction

Environmental education for children is still dominated by reductionist, information-oriented approaches [1–3]. Rather than emphasizing the complexity of environmental systems, many educators have been encouraged to break down environmental science concepts into small components that can be studied in isolation from each other. Although this reductionist approach may be quite effective in accomplishing certain important pedagogical goals, it may be insufficient in generating a complete understand of how real-world problems and solutions result from interactions within complex systems. While many holistic approaches to environmental education have been shown to be effective in real-world applications [4], few of them have directly focused on developing or assessing children’s “systems thinking” skills. The research in this paper introduces Environmental Dashboard (ED), a technology and approach that displays real-time community resource use, as a teaching tool. We explored the technology’s effect on students’ systems thinking skills, content retention, and feelings of efficacy.

### Systems thinking, described

“Systems thinking” is an intellectual framework that has been applied across a variety of disciplines to explain, organize, and address the integrated behavior of social, ecological and economic systems. It assumes that system dynamics result from interactions, feedback loops and chains of causality between system parts [5, 6]. Systems thinking is similar to what has been termed holistic thinking in its emphasis on whole-system dynamics and on the importance of interactions between components as determinants of whole-system behavior. However, systems thinking places more emphasis on analytical thinking and is also paired with a normative claim that it should improve decision-making skills [7]. For the purposes of the research described in this paper, we will use an operational definition of systems thinking as a way of perceiving the world that:

- recognizes systems as being comprised of and exhibiting properties that result from dynamically interacting parts,
- incorporates a refined understanding of levels of cause and effect, including indirect as well as direct consequences and relationships between parts,
- situates the systems thinker within the systems they are studying.

Related to decision-making, systems thinkers may have an increased sense of self-efficacy and group efficacy when it comes to addressing issues in their community. In a systems context, self-efficacy can be viewed as the degree to which an individual feels that she can use her personal knowledge of a system to take individual action and effect change in that system. Group efficacy is the degree to which an individual feels that she can work together with others to effect change [8].

### Content-specific vs. general systems thinking

In this research we make a distinction between “content-specific” systems thinking skills and “general” systems thinking skills. Content-specific systems thinking can be defined as the ability to remember and identify relationships and interactions between facts and concepts taught during a lesson. General systems thinking refers to the higher-level cognitive process of generalizing knowledge about patterns and relationships learned from studying one system in order to form ideas about another. It has been suggested that elementary education should focus on developing understandings of system components and basic relationships (i.e. content-specific skills) in order to create a base for the development of higher-order systems thinking skills (i.e., general systems thinking skills) in older students [9].

### Systems thinking in curricula

Recent research suggests that systems thinking can, in fact, be explicitly taught in a classroom setting [6, 10, 11]. A general conclusion from prior studies is that students do not automatically or naturally integrate information–they must be explicitly taught to understand the nature and importance of relationships. However, research to date has generally focused on older children and adults, and been primarily observational. Experts have recommended that systems thinking be explicitly taught and integrated into curricula as early as elementary school [9, 12]. This suggests a need for the development of age-appropriate educational materials and assessment tools for systems thinking.

### Environmental Dashboard (ED) as a tool for promoting systems thinking

**Fig 1:**
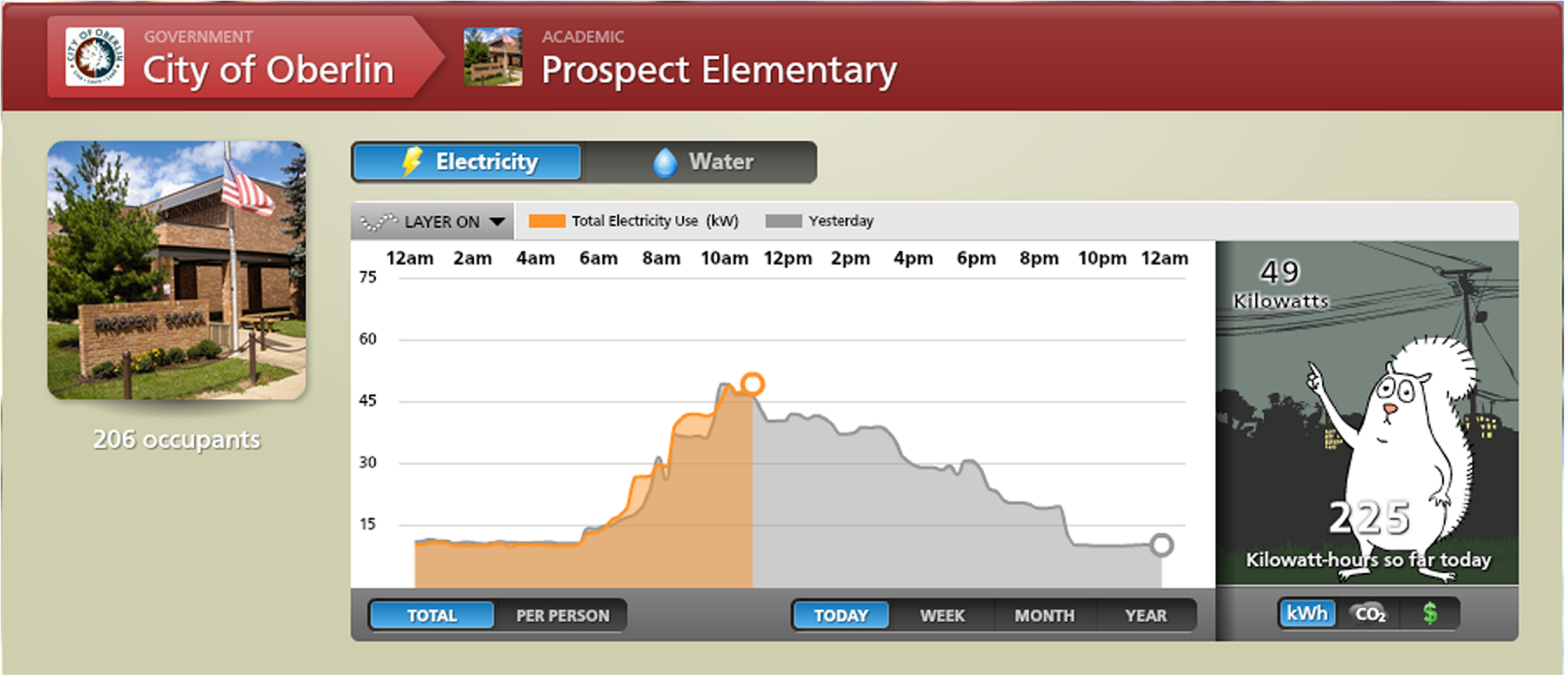
Building Dashboard. Designed to render information on resource use accessible and engaging to non-technical audience of different age levels. Current patterns are compared with past performance and among buildings. Character gauges animate in response to resource consumption to imbue quantitative data with emotional resonance (Reprinted from http://buildingdashboard.net/oberlincity/#/oberlincity/prospect under a CC BY license, with permission from Lucid Design, original copyright 2014).

**Fig 2:**
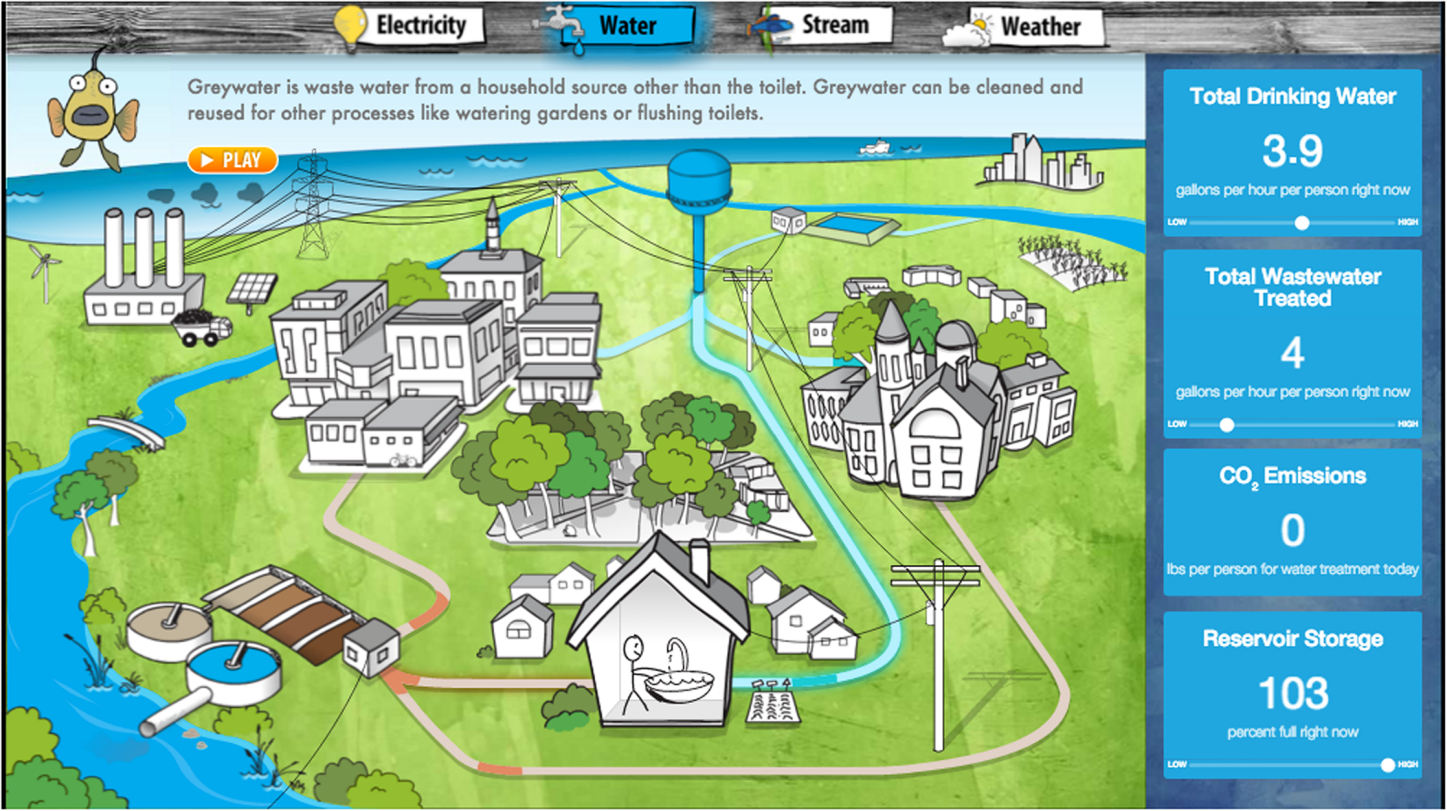
City-Wide Dashboard. “Flash” Energy Squirrel narrates the dynamic story of current flows of water and electricity and environmental conditions in whole communities. Goal is to situate individual decision-making within community context.

Environmental Dashboard is a sociotechnical feedback technology that monitors and displays real-time resource flows through whole cities and in individual buildings and combines this with images and text [8, 13]. Environmental Dashboard is comprised of three distinct, but interconnected components:

1. Building Dashboard–Time-series graphs that indicate real-time use of electricity and water in individual buildings. An “empathetic character” exhibits different behavior depending on relative levels of resource consumption (Fig 1 courtesy of Lucid®).
2. City-Wide Dashboard- An animated representation of the entire community, which illustrates real-time flows of electricity and water, and displays data on local environmental conditions such as water quality (Fig 2).
3. Community Voices—Photographs paired with quotes from community residents that highlight a locally-focused definition of sustainability and a commitment to the environment.

In combination, these three elements are currently accessible to the public through digital signage–ED is displayed on computer monitors hung in public places throughout the community such as schools, storefronts and libraries–and through a website (www.environmentaldashboard.org). The overall goal of ED is to engage, educate, motivate and empower change in thought and behaviors in order to benefit the environment and build community. (Building Dashboard is currently a commercial product made available by www.luciddesigngroup.com, Citywide Dashboard and Community voices are in advanced stages of development with the intent to be provided as shareware).

We expected ED to be a useful technology for promoting systems thinking for several reasons. First, the combined monitoring of resource use in individual buildings and throughout the whole community is intended to build an understanding of how parts (buildings) make up a whole (the entire city). Second, real-time display of data allows for the exploration of cause-effect relationships between consumer activities and resource consumption. Third, the featured words and images from community members in the Community Voices component helps situate the viewer within the ecological, social, and economic systems of their community. A prior study showed that, among college students, the City-Wide Dashboard component enhanced several aspects of systems thinking [8].

We also anticipated that ED would be a useful tool for promoting knowledge acquisition and retention in children. Real-time data provided by the Building Dashboards and City-Wide Dashboards paired with the Community Voices slides serve to connect classroom content with real-world problems and situate children’s learning in the context of their community. Research indicates that such real-world context can increase students’ self-efficacy and interest in the topic, and result in higher retention of knowledge and skills [14, 15]. Similarly, the sense of surprise and mystery stimulated by real-world educational discovery has been shown to elicit emotional responses that have biochemical connections to memory, further supporting content retention [16].

### Research questions

This research aimed to evaluate ED as a tool for teaching systems thinking skills and for supporting the acquisition and retention of content knowledge in 4^th^ and 5^th^ graders. It examined these effects when students were allowed to passively view ED, and when ED was integrated into curricula. Specifically this research addressed the following questions:

- Does passive exposure to ED increase general systems thinking?
- Are classroom exercises using ED effective for teaching content knowledge in primary schools?
- Are classroom exercises using ED effective for teaching either content-specific or general systems thinking skills to children?
- Does active engagement with ED support a sense of either self or group efficacy in addressing environmental issues?

## Methods

### School context

Research took place within Prospect Elementary, a public school for grades 3–5 (children age 8–10 years old). Prospect has a student enrollment of approximately 210 with 20 per classroom. The Oberlin School district follows the International Baccalaureate (IB) curriculum, which emphasizes civic engagement, community wellbeing and sustainability education [17]. Like all public schools in Ohio, the curriculum incorporates content and skills that must meet a variety of state-wide standards. Prospect is economically, racially and culturally diverse (e.g. 50% of students qualify for economically subsidized lunch program and 49% are non-white).

### Treatments

There were two ED treatments conducted in Prospect as part of this research. In a *passive exposure* treatment, a digital sign with ED content was installed in a well-travelled hallway and surveys of all students in the school were conducted before and after several months of exposure to assess impact on systems thinking. In an *active engagement* treatment, ED was incorporated into lessons on electrical circuits in a subset of classrooms, and a controlled experiment was conducted to assess impacts on systems thinking and content retention.

### Passive ED exposure

In Fall 2012, equipment was installed in Prospect to monitor whole-building electricity and water consumption in real-time. An ED screen was installed in the school’s main hallway. The screen displayed a rotating sequence of the school’s Building Dashboard (Fig 1), the City-wide Dashboard (Fig 2), and Community Voices content. All Prospect students were surveyed before the installation of the screens, and again at the end of the year, to measure changes in systems thinking skills. Students in a comparable elementary school in a neighboring county served as a control group and were given the same surveys but with no exposure to ED. As of the writing of this paper, the ED screen in Prospect remains turned on in the school, and all Prospect students continue to passively experience ED.

#### Active ED engagement

In Winter 2013–2014, a unit focused on electricity that is part of the normal curriculum and is required as part of Ohio State learning standards was taught in the classes of three 4^th^ and three 5^th^ grade teachers. The school system identified two 4^th^ grade and one 5^th^ grade teachers, to incorporate three 30–45 minute lessons that used ED to teach and support key aspects of this unit. All six teachers agreed to administer an assessment to their students before and after the unit to measure knowledge acquisition and retention and systems thinking skills. Classrooms in the treatment group incorporated ED into lessons on three different days. The other teachers (control group) used their existing lessons with no reference to the dashboard.

We worked with Prospect teachers to select applicable learning standards and goals from the Electricity Unit to be addressed in the ED lessons, including newly revised Ohio State Learning Standards. These lessons used the ED technology to emphasize relationships between content required in standards and the flows of electricity through the school and city and the larger environmental implications and connections associated with these resource flows. Teachers in the ED Treatment group still taught the majority of lessons associated with the electricity unit. In order to maximize consistency in delivery researchers guest-taught the three lessons focused on using ED.

### Data sources and analysis

Three sources of data were used to assess ED’s effectiveness: general systems thinking surveys, electricity unit content tests, and qualitative feedback and observation from teachers and researchers. A 2 (Time: pre vs. post-treatment) x 2 (Group: dashboard treatment vs. control) mixed model analysis (ANOVA) was run to assess ED’s impact on systems thinking skills, self and group efficacy, and content acquisition. Signed parental consent was obtained for each student who participated in any surveys associated with this research. The research protocol was reviewed and approved by the Institutional Review Board of Oberlin College, which ensures that all research involving human subjects meets high ethical standards.

#### Source 1: General system thinking surveys (2012–2013 and 2013–2014)

We administered a systems thinking pre-treatment survey in Fall 2012 to all Prospect 4^th^ and 5^th^ graders and to students in the same grade in a control school in Bexley Ohio that also uses the IB curriculum. We administered a post-treatment survey again in Spring 2013 in Prospect after the passive engagement ED treatment was completed. The same survey was also deployed in the Bexley school in Spring 2013. Questions were designed to assess three distinct dimensions of systems thinking: perception of ecological, social and economic systems; connectedness with nature [18]; and sensitivity to indirect consequences and causal chains [19]. We administered the same pair of surveys again in Prospect (not Bexley) in the 2013–2014 school year. The post-treatment survey deployed in Prospect was administered after the electricity unit (active ED engagement).

#### Source 2: Electricity unit content tests (Winter, 2014)

In Prospect elementary, pre-tests were administered before the unit and post-tests after the unit in both control and treatment groups to assess differences in content retention, content-specific systems thinking skills, and perceptions of efficacy. Variables were defined, quantified and assessed as follows:

1. Content retention—Retention was measured using eight separate questions, mostly in multiple-choice format, which included graph interpretation, unit identification, and understanding of climate change. An aggregate content retention score was used which equaled the number of correct answers generated by each student.
2. Content-specific systems thinking—This question was intended to allow students to illustrate systems thinking skills in identifying indirect consequences related to electricity use. The question asked, “How could turning on a light in Oberlin eventually affect a Polar Bear at the North Pole?” Students were, in effect, asked to identify a chain of causation based on their knowledge of electricity and climate systems. Students received one point for each correct causal link they identified. In order to avoid potential bias in interpreting this data, the research assistant interpreting data was blind to whether data were from control or treatment condition.
3. Self-efficacy & group efficacy—Students were asked to list all the things they could do on their own in their school to save electricity, and then to list the ways they could work together with other students and adults to accomplish this goal. Scores were given by counting the number of solutions each student provided.

#### Source 3: Qualitative feedback & observation

All active ED engagement treatment group teachers were asked to fill out a form after the ED lessons that solicited their thoughts, observations, and suggestions related to the lesson. The researcher who administered the lessons also contributed qualitative observation on their experience.

## Results

### Effect of passive exposure on general systems thinking

In order to assess the impact of passive exposure to the digital signage that featured ED content we compared Fall 2012 and Spring 2013 survey responses between the Prospect students, who had been exposed to ED, and the Bexley students who had not been exposed. Specifically, we ran a 2 (Time: pre vs. post-treatment) x 2 (Group: dashboard treatment in Prospect vs. Bexley control) mixed model analysis (ANOVA). We assessed all three dimensions of systems thinking on the surveys and found no main effects of passive exposure to the dashboard or interactions with Group, all *p’*s > .296.

### Effects of Active ED engagement

We first assessed data for preexisting differences between students in the different classes for all variables measured. ED treatment and control groups did not differ in the pre-treatment scores on content retention, content-specific systems thinking, or general systems thinking, all *p*’s > .159. However, prior to the experiment, students in the control condition scored marginally higher than those in the treatment condition in our measure of self-efficacy, *t*(82) = 1.75, *p* = .084, and significantly higher on group efficacy, *t*(82) = 2.01, *p* = .047.

Since it is possible that 4^th^ and 5^th^ grade students might respond differently, we included grade in our analysis of the effects of active ED engagement. Specifically, for each variable examined, we ran a 2 (Time: pre vs. post-treatment) x 2 (Group: dashboard treatment vs. control) x 2 (Grade: 4^th^ vs. 5^th^) mixed model analysis of variance (ANOVA). Although grade was included in all analyses below, there was only one significant effect of grade; we observed a grade x time interaction on content retention. Since this particular interaction does not affect interpretation of the effect of active ED engagement that we are interested in evaluating, grade effects are not discussed further.

A summary of main effects, interactions, means, and standard deviations is reported in Table 1. For the interactions testing our main hypothesis (time x group interaction), we also calculated an effect size using Cohen’s *d*, a standardized measure of the difference between means. A Cohen’s *d* between 0.5 and 0.8 is considered to be a medium-size effect; above 0.8 is considered to be a large effect [20].

**Table 1:** Main effects, interactions, means and (standard deviations).

Dependent Variable	Main Effect (time)			Interaction Effect (time x group)			Time 1		Time 2	
							Control	Dashboard	Control	Dashboard
	df	F	*p*	df	F	*p*	M (SD)	M (SD)	M (SD)	M (SD
Content retention	1, 67	74.32	** .000	1, 67	9.61	** .003	4.97 (2.33)	4.35 (2.04)	6.14 (2.49)	7.65 (1.86)
Content-specific systems thinking	1, 76	59.69	** .000	1, 76	16.66	** .000	0.64 (1.09)	0.56 (1.07)	1.62 (1.80)	3.07 (1.78)
General systems thinking	1, 73	2.15	.147	1, 73	1.39	.243	3.78 (1.16)	4.11 (1.14)	3.94 (1.00)	3.92 (1.31)
Self-efficacy	1, 76	0.13	.724	1, 76	6.42	** .013	3.44 (3.59)	2.61 (1.14)	2.74 (1.14)	3.22 (1.35)
Group efficacy	1, 76	1.86	.176	1, 76	3.97	* .050	3.15 (2.99)	2.10 (1.39)	2.82 (2.05)	3.29 (1.63)

** = results significant at 0.001 level

* = results significant at 0.05 level

### Content retention

The content retention scores revealed a significant main effect of Time; both the control and ED treatment groups improved their scores on tests measuring knowledge of the subject taught between pre-test (Time 1) and post-test (Time 2), *F*(1,67) = 74.32, *p* < .001. There was a significant Time x Group interaction, *F*(1,67) = 9.61, *p* = .003. A planned contrast indicated that the ED treatment group retained significantly more content than the control group from Time 1 to Time 2, *t*(69) = 4.38, *p* < .001, *d* = 1.033.

### Content-specific systems thinking

A significant main effect of Time was evident in content-specific systems thinking; both the control and ED treatment groups improved their test scores between Time 1 and Time 2, *F*(1,76) = 59.69, *p* < .001. There was also a significant Time x Group interaction, *F*(1,76) = 6.66, *p* <. 001. A planned contrast indicated that the ED treatment group improved significantly more than the control group from Time 1 to Time 2, *t*(78) = 3.88, *p* < .001, *d* = 0.871.

### General systems thinking

We found no significant differences in general systems thinking score between the pre and post-treatment surveys, and saw no significant effect of ED treatment vs. control group, all *p*’s > .671.

### Self-efficacy

The self-efficacy measure revealed no significant main effect of Time, *F*(1,76) = 0.13, *p* = .724; on average, students did not list more things they could personally do to conserve electricity at Time 2 than at Time 1. However, this main effect was qualified by a Group x Time interaction, *F*(1,76) = 6.42, p = .013. A planned contrast indicated that the control group decreased in number of listed ideas marginally significantly, whereas the ED treatment group generated significantly more ideas at Time 2, *t*(78) = 2.84, *p* = .006, *d* = 0.631.

### Group efficacy

The group efficacy measure revealed no significant main effect of Time, *F*(1,76) = 1.86, *p* = .176; on average, students did not list more ways they could work together to conserve electricity at Time 2 than at Time 1. However, this main effect was qualified by a Group x Time interaction, *F*(1,76) = 3.97, *p* = .050. A planned contrast indicated that the control group decreased in number of listed ideas non-significantly, whereas the ED treatment group generated significantly more ideas at Time 2, *t*(78) = 2.70, *p* = .008, *d* = 0.602.

## Discussion

We were pleased to find that students in the classes that were actively engaged with ED lessons exhibited significantly greater improvements in both content retention and content-specific systems thinking than students in the control classes. However, some caution is warranted in interpreting these results. For instance, it is possible that factors such as the excitement of having a guest teaching in the classroom could have impacted students’ learning. Further, because classrooms were not randomly assigned to the treatment groups, we cannot entirely rule out the possibility that other differences between classes might have impacted performance. Nevertheless, since the researchers who guest taught ED lessons were not experienced teachers, we are inclined to believe that any bias resulting from researcher’s participating in teaching was towards less effective teaching and learning rather than towards the enhanced learning observed. We therefore interpret the results as supportive of the conclusion that ED provides an effective tool for helping students learn and remember content, and integrate new information into an understanding of larger systems.

Students who were actively engaged with ED lessons exhibited significantly higher perceptions of self-efficacy and group efficacy than students in the control classrooms. In addition to the results of the present study, we observed additional evidence supporting the conclusion that ED increases sense of efficacy in addressing environmental issues. Following the research described in this paper, ED displays were installed in all four Oberlin public schools. To initiate this installation, we organized a competition among the schools; students in the schools engaged in a two-week long competition to see which school could reduce their electricity use by the largest percentage relative to baseline period prior to the competition. Prospect Elementary, the school with two years of exposure to ED, won 1^st^ place with a reduction of 37% (the High School came in 2^nd^ place with a 22% reduction). Among other things, Prospect students convinced administrators to keep the main office lights off during the day, and to keep light levels in the cafeteria low during the competition. These observations are consistent with the notion that a longer exposure and greater experience with ED result in enhanced motivation and/or enhanced understanding of how to best reduce electricity consumption in a facility.

Although active engagement with ED increased content-understanding and content-specific systems thinking skills, we did not observe increases in general systems thinking in response to this treatment. Likewise, we were not able to detect a significant change in children’s general systems thinking skills as a result of several months of passive exposure to digital signage featuring ED in the hallway. While it may well be that there really is no impact of either active or passive exposure on general systems thinking, we hesitate to draw broad conclusions for a number of reasons. For example, the survey questions we used to measure general systems thinking were derived from measures designed for adults; it is possible that measures designed with greater attention to the developmental state of children would have been more sensitive. In the case of our assessment of passive exposure to digital signage, five months passed between the pre-test and the post-exposure test during which many things changed in students’ lives and learning. The signal generated by exposure to a digital sign in the school was likely weak relative to these other factors. It is also possible that passive exposure (a couple of views per day while passing digital signs in the hallway) simply requires more time to change thought. We believe that further research is warranted; future experiments should develop more sensitive and age-appropriate metrics for assessing general systems thinking and should be conducted over longer periods of time. All three teachers in whose classes ED lessons were deployed indicated that they felt the ED lessons increased students’ enthusiasm towards the topics presented in the classrooms and agreed that the ED lessons fit very well with the IB curriculum and state standards. The Dashboard related lesson is now used by all teachers in the 4^th^ grade where the circuit unit is now taught.

## Implications

High stakes-testing and standardized curricula have been centerpieces of policymaking in American public education for several decades now [21–23]. The pressure to cover required curricular standards means that teachers don’t always have time to develop strategies for teaching more abstract skills or concepts such as systems thinking that are not directly incorporated into curricular standards or testing. The research described in this paper suggests that technology such as Environmental Dashboard, which places a strong emphasis on teaching systems thinking skills, can be used to enhance content retention relevant to standards while concurrently promoting content-specific systems thinking skills, and a sense of self and group efficacy.

A goal of our research group has been to make ED technology accessible and useful to other communities. Assuming that further research continues to document curricular value, it will be important to develop a means of disseminating new curricular resources on effective use as they are developed. In her review of 30 years’ worth of environmental education research, Lucie Sauvé [24] posed the question, “considering the state of our world, would it be unethical to conduct environmental education without focusing on concrete problem-solving?” To that end, a significant amount of scholarship in the past decade has explored the question of how best to educate students to serve as agents of social and environmental change [4, 25–27]. That research has revealed that the understanding and skills that students develop early on has the potential to influence behavior throughout their lifetime. What they learn may also influence the thinking and behavior of adults in their lives [28]. The research described in this paper suggests that Environmental Dashboard may be a useful tool for building systems thinking skills that will be important for addressing the range of increasingly complex challenges that this generation will face.
